# Anterior Abdominal Wall Pseudohernias After Anatomic Lung Resection: Incidence and Risk Factors

**DOI:** 10.1016/j.atssr.2025.02.002

**Published:** 2025-02-28

**Authors:** Andrew Behrmann, Blake Wojciechowski, Chase Schlesselman, Jussuf Kaifi, Sebastian Wiesemann

**Affiliations:** 1 Division of Cardiothoracic Surgery, Department of Surgery, University of Missouri School of Medicine, Columbia, Missouri

## Abstract

**Background:**

Thoracic surgery can damage intercostal nerves and cause muscular atrophy and bulging of the anterior abdominal wall (pseudohernia). This pilot study investigated the incidence of and risk factors for development of pseudohernias after anatomic lung resection in either robotic video-assisted thoracoscopic surgery (R-VATS) or thoracotomy cases.

**Methods:**

A retrospective cohort analysis of 319 patients undergoing either R-VATS or thoracotomy for anatomic lung resection at a single institution from 2017 to 2021 was performed to determine pseudohernia incidence rates and possible risk factors.

**Results:**

Only patients who underwent R-VATS had pseudohernias, with an incidence rate of 7.6%. Readmission within 30 days of operation was higher in patients with pseudohernias (*P* = .02). Cryoablation at or below the seventh intercostal space was significantly correlated with pseudohernia development (*P* = .04). Diabetes trended toward increasing the risk for pseudohernias (*P* = .05). Acute and chronic pain scores were higher in patients with pseudohernias.

**Conclusions:**

Robotic surgery and cryoablation are associated with an increased risk of pseudohernias, and the incidence may be higher than previous case reports suggest. Possible explanations are decreased tactile feedback, larger-diameter trocars, and lower intercostal access levels, leading to thoracoabdominal nerve damage. Understanding the incidence and risk factors for pseudohernias may inform surgical practices to improve patient outcomes and quality of life.

## Visual Abstract

**Figure:**
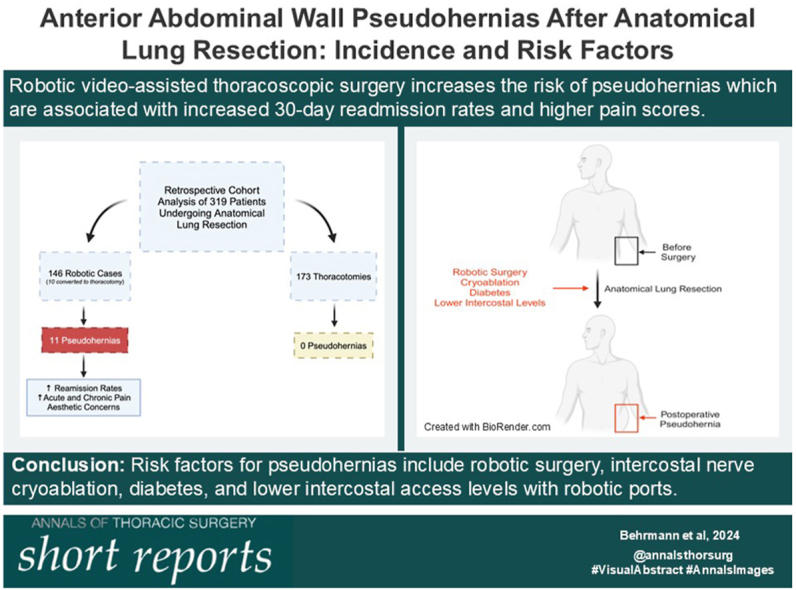

**In Short**

▪ Robotic surgery, diabetes, and cryoablation increase the risk of anterior abdominal wall denervation and pseudohernias.
▪ Pseudohernias are associated with short- and long-term pain scores and are aesthetically concerning for patients.

Thoracoabdominal nerve (T7-T11) damage can cause abdominal wall paralysis and bulging (pseudohernia). Trocars from robotic video-assisted thoracoscopic surgery (R-VATS) can damage intercostal nerves (Figure1A) and lead to abdominal wall atrophy and bulging (Figure1B). This complication can be painful and aesthetically concerning. Pseudohernias have previously been reported after thoracic surgery and are associated with thoracotomies and minimally invasive procedures alike.^1^, ^2^, ^3^, ^4^, ^5^, ^6^, ^7^ Some investigators have hypothesized that pseudohernia development is rare, whereas others claim that pseudohernia is an underreported complication.^6^^,^^7^ This pilot study was a cohort-level investigation of the overall incidence of pseudohernia development after R-VATS and thoracotomy procedures for anatomic lung resections, risk factors associated with pseudohernia development, and the effect of pseudohernias on pain scores.

**Figure 1:**
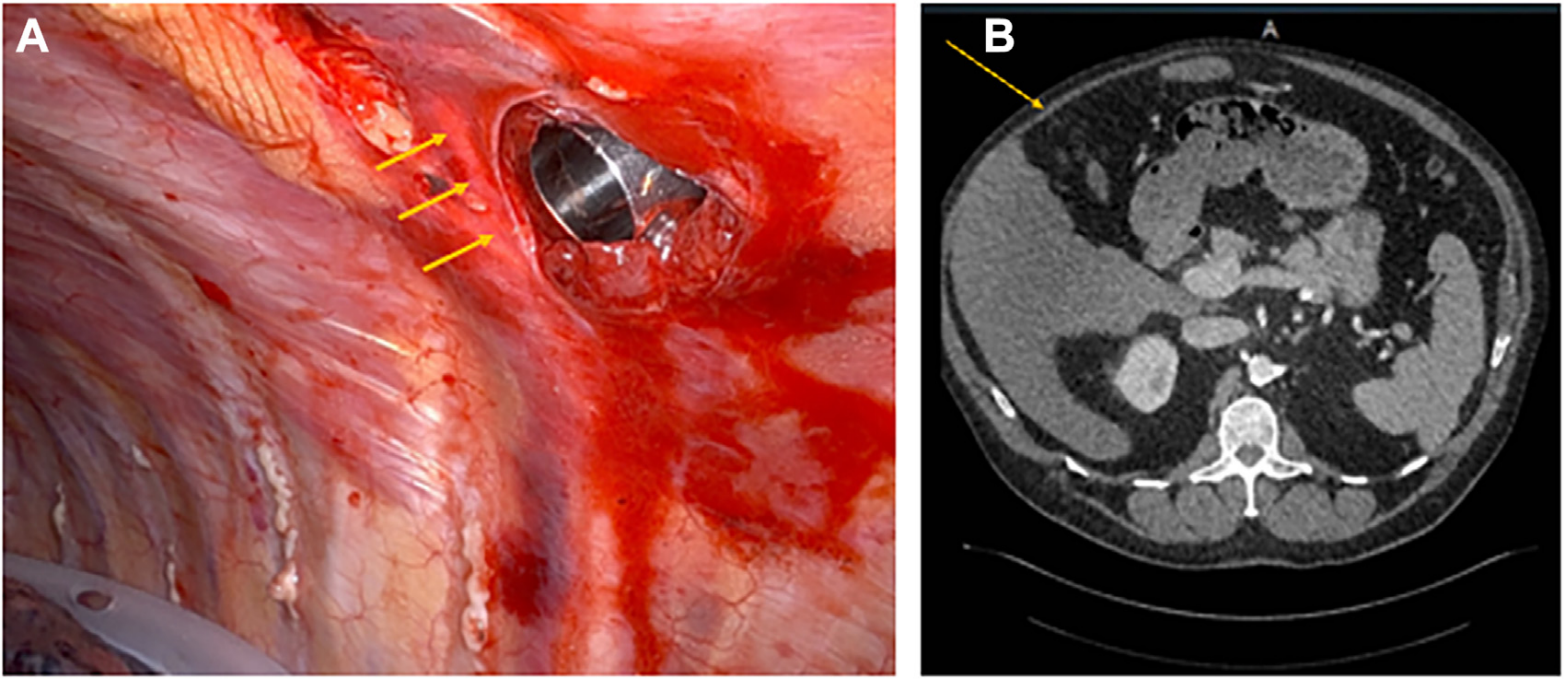
(A) Image of the trajectory of the seventh intercostal nerve and its proximity to bruising and tissue damage around the port site (arrows). (B) Sample computed tomographic scan showing anterior abdominal wall atrophy (arrow) from a patient with a pseudohernia.

## Patients and Methods

### Data Collection

This project received approval from the University of Missouri Institutional Review Board (IRB) on July 13, 2022 (IRB number 2091939). Data were retrospectively collected from 319 patients (146 R-VATS, 173 open operations) (Table 1) who underwent anatomic lung resections at a single center. Follow-up charts up to 1 year postoperatively were reviewed for pseudohernia development, diagnosed clinically by a unilateral bulge on the surgical side while the patient was standing. Analyzed variables included comorbidities (diabetes, smoking, body mass index, narcotic use), intercostal trocar or incision levels, type of resection, bupivacaine nerve blocks, and cryoablation levels. Short-term (postoperative days 0-7) and long-term (0-6, 7-12, and 13-18 months) pain scores were assessed. Patients who underwent R-VATS that was converted to thoracotomy or who had multiple thoracic surgical procedures were excluded from pain analyses. Readmission, mortality, and pseudohernia duration were also evaluated.

**Table 1:** Patient Characteristics

Characteristic	R-VATS (n = 146)	Thoracotomy (n = 173)	*P* Value
Female	91 (62.3)	92 (53.2)	067
Age, y	64.5 ± 8.5	63.8 ± 9.7	.352
Body mass index, kg/m^2^	27.9 ± 6.7	27.9 ± 7.0	.745
Cryoablation	70 (47.9)	150 (86.7)	<.001
Cryoablation below T7	59 (84.3)	60 (40.0)	<.001
Smoker	125 (85.6)	138 (79.8)	.832
Type 2 diabetes	24 (16.4)	25 (14.5)	.809
Preoperative opioids	37 (26.1)	29 (16.7)	.081
Postoperative opioids	78 (53.4)	84 (48.5)	.924
Liposomal bupivacaine	86 (58.9)	66 (38.2)	.002
30-d readmission rate	18 (12.3)	23 (13.3)	.657
30-d mortality rate	3 (2.1)	2 (1.25)	.526
Duration of surgery, min	278 ± 99	256 ± 101	.155

Values are n (%) or mean ± SD.

R-VATS, robotic video-assisted thoracoscopic surgery.

### Statistical Analysis

A group (pseudohernia vs no pseudohernia)–by-time repeated measures analysis of variance (ANOVA) was performed to determine short- and long-term differences in pain between R-VATS–treated patients who did or did not experience pseudohernias. Long-term pain scores in patients who underwent R-VATS (excluding patients with pseudohernias) and thoracotomies were similarly analyzed, stratified by the use of cryoablation. A Bonferroni post hoc test was used to identify differences between groups for all ANOVAs. A χ^2^ test of independence was performed on categorical outcomes. Considering thoracoabdominal innervation (T7-T11), the use of cryoablation and cryoablation below the seventh intercostal level were treated as separate binary variables for χ^2^ analysis. Statistical significance was assigned to *P* values <.05.

## Results

### Incidence of Pseudohernias and Their Effect on Readmission Rates

Pseudohernias developed only in patients who underwent R-VATS procedures. Of the 146 R-VATS procedures, 11 pseudohernias developed (7.6%). Two of the 11 pseudohernias developed in patients who underwent R-VATS procedures that were converted to thoracotomies. Characteristics of the patients with pseudohernias are displayed in Table 2. R-VATS–treated patients who had pseudohernias were more likely to be readmitted to the hospital within 30 days of their operation (36.4% vs 10.4%; *P* = .01) with no specific trends in the reason for readmission (Table 2).

**Table 2:** Characteristics of Patients With Pseudohernias

Age, y	Sex	Type of Resection	ICS Trocar Level	Cryoablation Level	BMI, kg/m^2^	Diabetes	Readmission Reason	Last Documented Persistence of Pseudohernia, mo
63	F	Lobectomy	8, 9, 11	N/A	20	No	Pneumothorax	3
61	F	Lobectomy	7, 8, 9, 11	T3-T9	47	Yes	Pneumonia	Unresolved
58	F	Lobectomy	8	N/A	32	Yes	N/A	<1
60	M	Lobectomy	6, 7, 9, 10	T4-T10	32.1	No	N/A	Unresolved
64	M	Lobectomy	6, 7, 9, 10	T4-T10	23	No	Pain	Unresolved
67	M	Lobectomy	6, 7, 9, 10	T5-T10	22.1	No	Empyema	1
61	F	Lobectomy	6, 7, 9, 10	T4-T9	35	No	N/A	6
65	F	Lobectomy	7, 8, 10, 11	T4-T10	25.8	No	N/A	<1
67	F	Lobectomy	7, 8, 9	T5-T10	31.2	Yes	N/A	12
67	F	Segmentectomy^a^	…	N/A	27	No	N/A	13
58	M	Lobectomy^a^	8	T3-T9	32	Yes	N/A	12

BMI, body mass index; F, Female; ICS, intercostal space; M, Male; N/A, not applicable.

a Robotic video-assisted thoracoscopic surgery that was converted to thoracotomy.

### Risk Factors Associated With Pseudohernia Development

Pseudohernia risk was unaffected by nerve blocks (*P* = .14), opioid use (*P* = .24), or body mass index (*P* = .48). The use of cryoablation in R-VATS cases trended toward increasing the risk of pseudohernia development (8 of 70 patients who underwent cryoablation, 8 of 11 pseudohernias; *P* = .09). Undergoing cryoablation at or below the seventh intercostal level showed an increased risk of pseudohernia development in R-VATS cases (8 of 59 patients who underwent cryoablation below T7, 8 of 11 pseudohernias; *P* = .04). Type 2 diabetes also trended toward increasing the risk of pseudohernia development (*P* = 0.05).

### Pain Scores in Patients Who Underwent Robotic Video-assisted Thoracoscopic Surgery With and Without Pseudohernias

**Figure 2:**
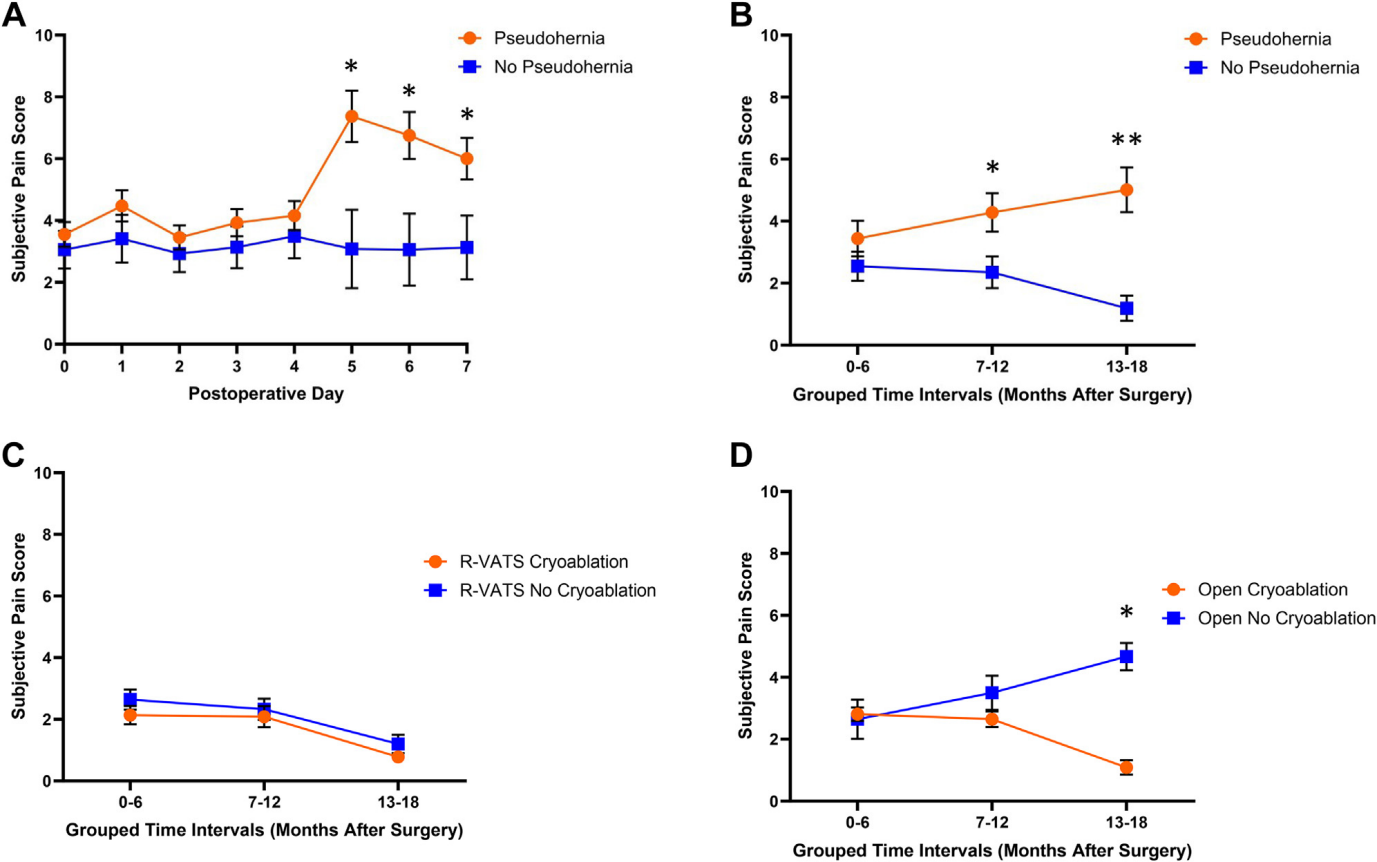
Subjective (A) short-term and (B) long-term pain scores in patients undergoing robotic video-assisted thoracoscopic surgery (R-VATS) who did and did not have pseudohernias. Long-term pain scores of patients undergoing (C) R-VATS and (D) open procedures with and without cryoablation. Analyzed by group-by-time repeated measures analysis of variance.

Figure 2A shows the R-VATS–treated patients who experienced pseudohernias had consistently higher pain scores throughout the first week when analyzed by group-by-time ANOVA (Figure 2A) (main effect of pseudohernias; *P* < .01). Group-by-time ANOVA of 6-month intervals out to 18 months postoperatively showed higher long-term pain scores (Figure 2B) (main effect of pseudohernias; *P* < .05) for patients with pseudohernias and significantly greater levels of pain 7 to 12 months (*P* < .05) and 13 to 18 months (*P* < .01) postoperatively. Cryoablation did not affect long-term pain scores in R-VATS cases (Figure 2C), but it was associated with lower pain scores in patients who underwent thoracotomies (Figure 2D) (main effect of cryoablation; *P* < .05).

## Comment

The thoracoabdominal nerves (T7-T11) supply motor innervation to the anterior abdominal wall. It is common in some surgical practices to place ports at these intercostal levels during R-VATS procedures, thereby increasing the risk of nerve injury (Figure 1). In this study, the 7th to 11th intercostal spaces were accessed in only 1.25% of thoracotomy cases, but at least 1 port was placed within this range in 100% of R-VATS cases. Consequently, thoracoabdominal nerve damage was more likely during robotic procedures, particularly because robotic systems lack haptic feedback, thus potentially increasing tissue damage.

Our robotic approach traditionally involves placing a 12-mm robotic port near the costal margin and 3 additional 8-mm robotic ports approximately 4 fingers apart along the seventh intercostal space, sometimes extending to the eighth. Additionally, 12-mm AirSeal ports (ConMed) were positioned posterolaterally in the 10th or 11th intercostal space. Some port placements varied on the basis of anatomy and case requirements. For example, the anterior 12-mm port may have been placed in the sixth intercostal space, with 8-mm robotic ports spanning the seventh to ninth intercostal spaces more perpendicularly and the 12-mm AirSeal port sometimes positioned more anteriorly.

Cryoablation was performed using the AtriCure system, with extended-release bupivacaine administered for an initial nerve block. Cryoablation was applied at the conclusion of the procedure by using an AtriCure probe inserted through an 8-mm port, targeting intercostal nerves for 2 minutes, each starting at a temperature of −40 °C with a target of −70 °C. Some cryoablation manufacturers advise against cryoablation below the ninth intercostal space because of the risk of abdominal muscle bulging. The 49 of 70 R-VATS–treated patients who underwent cryoablation, and 8 of 11 patients with pseudohernias who underwent cryoablation had this procedure below the ninth intercostal space. Our group initially considered cryoablation-induced nerve damage as temporary and an acceptable tradeoff for pain relief. However, our findings demonstrated no significant benefit to patient pain but an increased risk of pseudohernia development, thus prompting us to discontinue cryoablation in robotic cases and restrict its use to above the seventh intercostal space in thoracotomies.

A study examining cryoablation for postthoracotomy pain syndrome observed that 23% of patients had pseudohernias as a complication of cryoablation.^8^ Our study reported that 73% of patients (8 of 11) who experienced pseudohernias had undergone cryoablation, and cryoablation below the seventh intercostal level was significantly correlated with pseudohernia development. Notably, none of the open surgical patients who underwent cryoablation below T7 had pseudohernias, although fewer underwent cryoablation at this level. These findings may suggest that the combination of lower port placement, cryoablation, posterior ports within narrower intercostal spaces, larger port sizes, and lack of tactile feedback during R-VATS inherently increases the risk of thoracoabdominal nerve damage. Diabetic neuropathy has also been implicated in pseudohernia development.^9^ Our study found that patients with type 2 diabetes showed a trend toward increased pseudohernia risk, possibly reflecting preexisting nerve damage exacerbated by surgical compression. Furthermore, pseudohernia development was associated with a higher 30-day readmission rate (36.4%) compared with the R-VATS cohort without pseudohernias (10.4%). Although no specific trends were observed in reasons for readmission among patients with pseudohernias, closer monitoring may help prevent readmissions.

To mitigate complications associated with robotic procedures, understanding risk factors specific to R-VATS is essential. Our practice has evolved on the basis of the results of this analysis, successfully reducing pain and pseudohernia rates through the following changes: (1) positioning robotic ports only along the seventh intercostal space, spaced 4 fingers apart, with the AirSeal port in the 10th posterolateral intercostal space; (2) pushing the robotic arm inferiorly toward the lower rib after docking to increase clearance for intercostal nerves; (3) using a 0° camera rather than a 30° camera for a steeper angle, thereby reducing intercostal nerve compression; (4) avoiding excessive posterior instrument movements to minimize nerve compression; and (5) extracting specimens through the anterior 12-mm port at the seventh intercostal space rather than through the posterolateral 10th intercostal space. These modifications have successfully mitigated complications, and we have observed no pseudohernia cases since their implementation.

Other potential strategies to limit intercostal nerve damage during R-VATS could include uniportal or transdiaphragmatic robotic approaches. Preoperatively, patients undergoing R-VATS or cryoablation should be informed of the pseudohernia risk. Management options for pseudohernias include abdominal binders to alleviate bulging sensations and pregabalin for neuropathic pain. Surgical repair, as described by Sharobaro and colleagues,^10^ involves reconstructing the abdominal wall with imbrication sutures and reinforcing it with mesh implants. However, surgical outcomes are limited, and neuropathic pain may persist despite repair.

### Study Limitations

With only 11 patients experiencing the primary outcome, including 2 patients whose procedures were converted from R-VATS to thoracotomy, this study may be underpowered for detailed analyses of risk factors and pain scores. The single-center retrospective design and reliance on electronic medical records for pseudohernia identification also limit generalizability.

### Conclusion

This cohort-level study reported the incidence of pseudohernias after anatomic lung resections and identified R-VATS as an independent risk factor, with a 7.6% incidence rate. Diabetes and cryoablation, particularly cryoablation below the seventh intercostal space, were additional risk factors, findings supporting a multifactorial model of pseudohernia development where multiple or severe insults to intercostal nerves contribute to anterior abdominal wall denervation, neuropathic pain, and pseudohernias. Larger, multicenter studies are needed to investigate these factors further and optimize preventive strategies.

## Declaration of Generative AI and AI-Assisted Technologies in the Writing Process

During the preparation of this work, the authors used ChatGPT-4 to cut words to fit the Short Reports format. After using this tool, the authors reviewed and edited the content as needed and take full responsibility for the content of the publication.
